# MiR-23a targets RUNX2 and suppresses ginsenoside Rg1-induced angiogenesis in endothelial cells

**DOI:** 10.18632/oncotarget.19489

**Published:** 2017-07-22

**Authors:** Xiao-Dong Wu, Ting Guo, Li Liu, Chao Wang, Kun Zhang, Han-Qiang Liu, Feng Wang, Wen-Dong Bai, Meng-Yao Zhang

**Affiliations:** ^1^ Department of Cell Biology, National Translational Science Center for Molecular Medicine, Fourth Military Medical University, Xi’an 710032, China; ^2^ Beijing Institute of Biotechnology, Beijing 100071, China; ^3^ Department of Clinical Immunology, Xijing Hospital, Fourth Military Medical University, Xi’an 710032, China; ^4^ Department of Nutrition and Food Hygiene, Fourth Military Medical University, Xi’an 710032, China; ^5^ Department of Stomatology, PLA General Hospital, Beijing 100700, China; ^6^ College of Life Science and Bioengineering, School of Science, Beijing Jiaotong University, Beijing 100044, China; ^7^ Clinical Laboratory Medicine Center, Xinjiang Command General Hospital of PLA, Urumqi 830000, China

**Keywords:** angiogenesis, ginsenoside Rg1, miR-23a, runt-related transcription factor 2, vascular endothelial growth factor

## Abstract

Rg1 is a predominant protopanaxatriol-type of ginsenoside found in Panax ginseng, and it has been shown to have anti-cancer effects in multiple types of cancer cells. However, Rg1 also induces the expression of proangiogenic factors, such as vascular endothelial growth factor (VEGF-A), in endothelial cells. Unfortunately, angiogenesis positively correlates with cancer development. In this study, we identified RUNX2 as a regulator of ginsenoside Rg1-induced angiogenesis for the first time. We found that RUNX2 was directly targeted and regulated by miR-23a. Additionally, miR-23a was shown to inhibit angiogenesis in both human umbilical vein endothelial cells (HUVECs) and in zebrafish. Furthermore, a decrease in RUNX2 expression resulted in translational repression of VEGF-A in HUVECs. Taken together, this study identified a MiR-23a/RUNX2/VEGF-A pathway in angiogenesis and shed light on the molecular mechanism of Rg1-induced angiogenesis. Thus, RUNX2 might be a potential therapeutic target in Rg1-mediated angiogenesis in cancer.

## INTRODUCTION

Traditional Chinese medicine has proven to be a promising route in the development of new drugs. Additionally, Panax ginseng (ginseng) has been considered to be one of the most renowned medicinal herbs and has been used for centuries for the treatment and prevention of various disorders [1]; however, its mechanism remains elusive [2]. The most recent studies show that most of the biological activities of ginseng can be attributed to a group of triterpenoid saponins known as ginsenosides [3–5]. According to their chemical structures, ginsenosides were classified into protopanaxadiol type (PPD-type) and protopanaxatriol type (PPT-type). PPD-type ginsenosides include Rb1, Rb2, Rg3, Rh2, and Rh3, and PPT-type include Rg1, Rg2, and Rh1[6]. Ginsenoside-Rg1 is a predominant PPT-type ginsenoside in ginseng that stands out from other candidates due to its positive biological activities, including immunity enhancement [7], neural protection [8], tissue regeneration [9], and anti-aging [10, 11]. Particularly, Rg1 demonstrated different functions in cancer cells and endothelial cells. For cancer cells, the Rg1-mediated anti-cancer effect has been widely acknowledged. Previous research has demonstrated that Rg1 may inhibit cancer cell proliferation and metastasis in multiple types of cancer by forming conjugates with carbon nanotubes (CNT) or suppressing the function of transforming growth factor-β1 (TGF-β1) [12, 13]. Research in endothelial cells has shown that Rg1 is involved in many complex interactions with angiogenic factors and extracellular matrix components [14, 15], which is beneficial for angiogenesis. Numerous studies demonstrate that Rg1 strongly induces proangiogenic factors, such as nitric oxide (NO) and VEGF-A in human umbilical vein endothelial cells (HUVECs). This induction functions through pathways dependent on phosphatidylinositol 3-kinase (PI3K)/Protein kinase B (Akt), b-catenin/T-cell factor, and hypoxia-inducible factor-1a (HIF-1a) [16, 17]. Although the function of Rg1 is different and relatively independent in cancer cells and endothelial cells, it has been establish that the angiogenic functions in endothelial cells can advance cancer development [18].

MicroRNA (miRNA) is a class of non-coding single stranded RNA molecules encoded by endogenous genes. They have a length of about 22 nucleotides and are associated with the regulation of post transcriptional gene expression in plants and animals [19, 20]. In current advancements in vascular development, the use of transcriptomics and miRNAs have come into focus, and several miRNAs associated with Rg1 have been identified [21]. However, a comprehensive understanding of angiogenic signaling cascades influenced by miRNAs still requires thorough investigation, and new Rg1-related miRNAs and their targets remain elusive.

Runt-related transcription factor 2(RUNX2) is a transcription factor belonging to the RUNX family that has been shown to play key roles in osteoblast differentiation [22, 23]. RUNX2 is also recognized as a marker of mesenchymal stem cells found in tumors [24] and has been proven to participate in various signaling pathways involved in cancer growth progression [25]. However, the mechanism of RUNX2 in angiogenesis is still unknown.

In this study, we identified RUNX2 as a regulator of Rg1-induced angiogenesis in endothelial cells, and we found that RUNX2 is directly targeted and regulated by miR-23a. Overexpression of miR-23a under Rg1 treatment caused a significant decrease in endothelial tube formation and cell motility. Furthermore, zebrafish embryos injected with miR-23a showed significant impediments in angiogenesis *in vivo*. Additionally, a MiR-23a/RUNX2/VEGF-A pathway was identified in endothelial cells, and this may provide new strategy for cancer therapy. In this study, we investigate the molecular mechanism of Rg1-mediated angiogenesis in endothelial cells, and we demonstrate a novel solution for anti-angiogenesis therapy by targeting RUNX2.

## RESULTS

### Ginsenoside Rg1 induces RUNX2 expression in HUVECs

**Figure 1:**
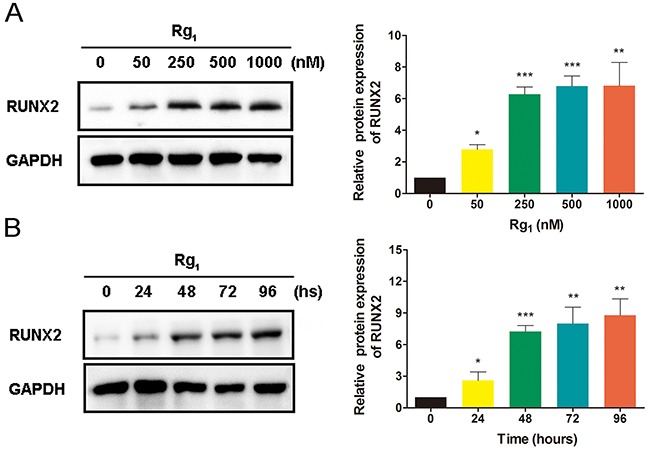
Ginsenoside Rg1 induces RUNX2 expression in HUVECs **(A)** Western blot analysis of RUNX2 in HUVECs after 96 hours stimulation by different dose of Rg1. **(B)** Western blot analysis of RUNX2 in HUVECs with 250 nM Rg1 stimulation at different time points. Data are plotted as mean ± SD of three separate experiments. (*P < 0.05;**P < 0.01;***P < 0.001).

As an important osteoblast differentiation marker, studies have shown that RUNX2 expression is upregulated by Rg1 in human periodontal ligament stem cells [26]. In our study, we assessed RUNX2 protein expression levels in HUVECs treated with or without Rg1 using western blot analysis with reference to GAPDH expression. We found that Rg1 increased RUNX2 expression in a dose-dependent manner (Figure 1A). Additionally, we tested the effects of Rg1 on RUNX2 protein expression from 0 to 96 hours. We also observed an increase in RUNX2 expression over time (Figure 1B).

### RUNX2 regulates VEGF-A expression under ginsenoside Rg1 treatment

**Figure 2:**
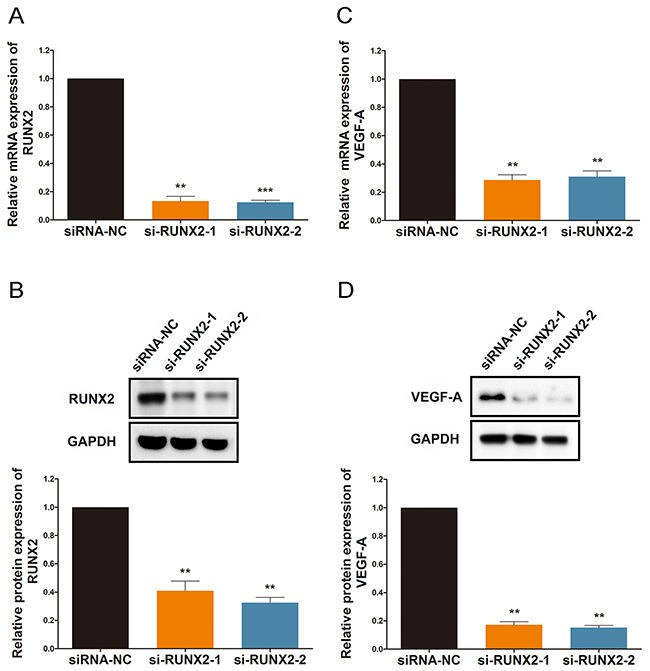
RUNX2 regulates VEGF-A expression under ginsenoside Rg1 treatment **(A and B)** Quantitative data and western blot analysis of si-RUNX2-1 and si-RUNX2-2 treated cells in HUVEC. Expression is relative to controls. **(C and D)** Quantitative data and Western blot of VEGF-A expression. Expression is relative to controls. Data are plotted as mean ± SD. (*P < 0.05;**P < 0.01;***P < 0.001).

The most thoroughly studied and most widely accepted regulatory factor of angiogenesis is vascular endothelial growth factor (VEGF-A) [27]. To determine a mechanism for the participation of RUNX2 in Rg1-induced angiogenesis, we studied the effect of RUNX2 on VEGF-A expression. To inhibit RUNX2 expression, we transfected HUVECs with si-RUNX2, and we were able to confirm high transfection efficiency using qRT-PCR and western blot analysis (Figure 2A and 2B). Inhibition of RUNX2 resulted in translational repression of VEGF-A in HUVECs (Figure 2C). As a result, there was a robust reduction in VEGF-A protein levels (Figure 2D).

### RUNX2 regulates cell viability and migration under ginsenoside Rg1 treatment

**Figure 3:**
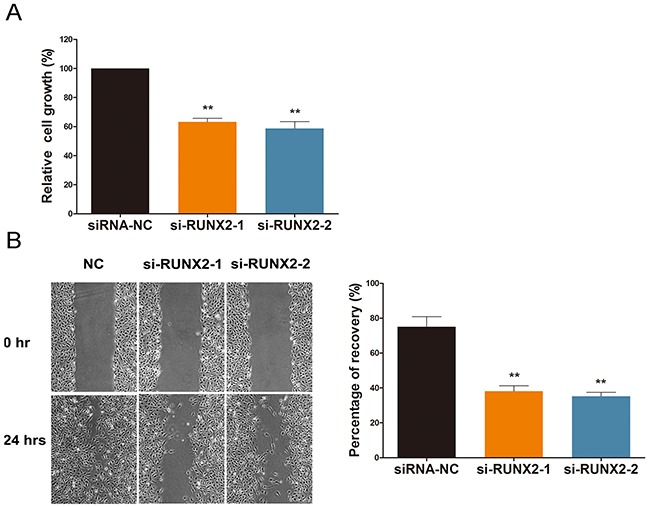
RUNX2 regulates cell viability and migration under ginsenoside Rg1 treatment **(A)** Cell proliferation percentage analysis after 24 hours treatment with si-RUNX2-1 or si-RUNX2-2. **(B)** Representative images demonstrating and statistical analysis of wound closure capacity of HEK-293 in si-RUNX2-1 or si-RUNX2-2 at 0h and 24h time points. Data are plotted as mean ± SD of three separate experiments. (*P < 0.05;**P < 0.01).

Cell viability and migration are imperative to angiogenesis [15]. Thus, we used HEK-293 cells and a cell migration assay to assess the motility of cells treated with ginsenoside Rg1. As shown in Figure 3, cells transfected with si-RUNX2 demonstrated reduced viability and motility when compared to the control group. We observed a decrease in wound recovery percentage from ~75% to ~40% (Figure 3). This suggests that RUNX2 facilitates cell motility and viability.

### RUNX2 is a regulator in ginsenoside Rg1-induced angiogenesis

**Figure 4:**
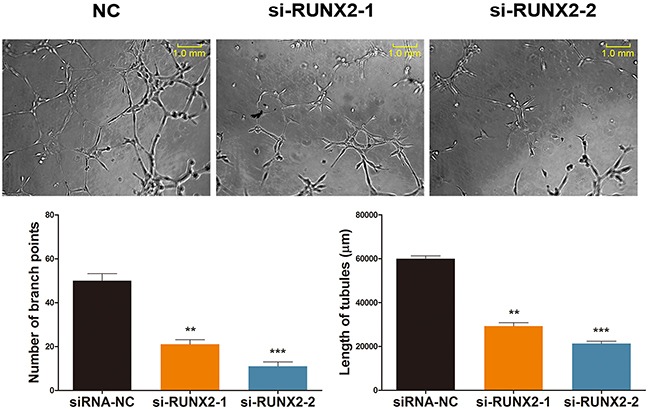
RUNX2 regulates ginsenoside Rg1-induced angiogenesis . Representative images demonstrating tube formation assay and statistical analysis. Cells were stimulated with 250 nM Rg1 and transfected with either si-RUNX2-1 or si-RUNX2-2, on day 3. Data are plotted as mean ± SD of three separate experiments. (**P < 0.01;***P < 0.001).

To further explore if RUNX2 is directly related to Rg1-induced angiogenesis, we performed a tube formation assay. This system mimics the reorganization of endothelial cells into a three-dimensional network during angiogenesis [28, 29]. Since the number of branch points reflects angiogenic activity in endothelial cells, we observed the capillary structures of HUVECs under various treatments. As shown in Figure 4, there was a significant decrease in endothelial tube formation when RUNX2 was inhibited under Rg1 treatment. The number of endothelial branch points was decreased by ~80% when compared with the control group. Additionally, the length of tubes was significantly shortened by RUNX2 inhibition (Figure 4).

### RUNX2 is directly targeted and regulated by miR-23a

**Figure 5:**
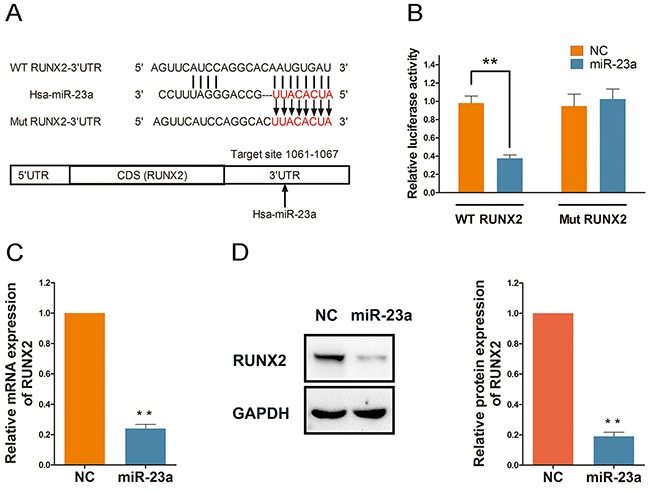
RUNX2 is directly targeted and regulated by miR-23a **(A)** TargetScan(http://www.targetscan.org/) and PITA were used to predict potential miRNA complementary targets. Potential target of miR-23a is partially complementary to the RNA sequence extending from nucleotide 1061-1067 within RUNX2 3’-UTR. **(B)** HUVECs were transfected with RUNX2 or Mut-RUNX2 3’-UTR luciferase reporter along with miR-23a mimic or control. **(C and D)** Quantitative data and Western blot analysis of RUNX2 in cells transfected with either miR-23a mimic or control. Protein expression is relative to controls. Data are plotted as mean ± SD of three separate experiments. (**P < 0.01).

To predict potential miRNAs that target RUNX2, we employed TargetScan and PITA online micro-RNA search programs. Our search results found that miR-23a was partially complementary to the RNA sequence of RUNX2 extending from nucleotide 1061–1067 (Figure 5A). Thus, we speculated that RUNX2 might be a target of miR-23a in Rg1-induced angiogenesis. To confirm this, we measured RUNX2 expression using a 3’-UTR luciferase reporter activity in a cell-based system. Compared to mutant RUNX2, miR-23a significantly reduced the wild-type RUNX2 3’-UTR reporter activity by approximately 60% (Figure 5B). Furthermore, qRT-PCR and western blot results demonstrated that miR-23a reduced RUNX2 expression through targeting of its 3’-UTR region (Figure 5C and 5D).

### Ginsenoside Rg1 reduces miR-23a expression

**Figure 6:**
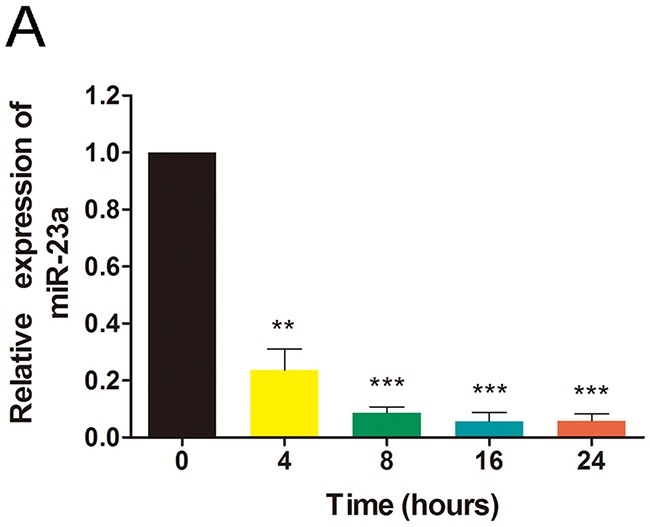
Ginsenoside Rg1 reduces miR-23a expression **(A)** Quantitative analysis of miR-23a in HUVECs after treatment with 250nM Rg1 for varying hours. Data are plotted as mean ± SD of three separate experiments. (**P < 0.01;***P < 0.001).

In order to determine whether Rg1 suppresses miR-23a expression in HUVECs, we employed qRT-PCR, using GAPDH as an internal control. As shown in Figure 6, the expression level of miR-23a decreased ~80% as early as 4 hours following Rg1 treatment. Moreover, 8 hours after Rg1 treatment, miR-23a expression level decreased more than 90%, and this suppression was sustained throughout 24 hours (Figure 6). These data indicate that the response of miR-23a to Rg1 treatment is quick but stable.

### MiR-23a regulates ginsenoside Rg1-induced VEGF-A expression

**Figure 7:**
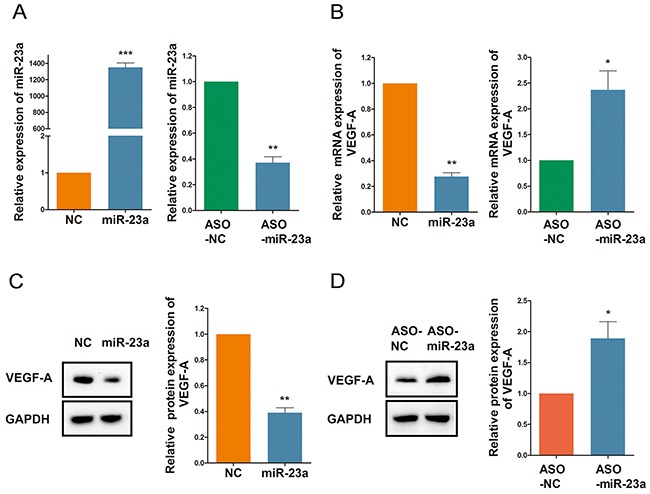
MiR-23a regulates ginsenoside Rg1-induced VEGF-A expression **(A)** Quantitative analysis of miR-23a expression following treatment of HUVECs with 50nM miR-23a or ASO-miR-23a. **(B)** Quantitative analysis of VEGF-A expression following treatment of HUVECs with 50nM miR-23a or 100nM ASO-miR-23a. **(C and D)** Western blot detection of VEGF-A expression following treatment of HUVECs with 50nM miR-23a or 100nM ASO-miR-23a. Data are plotted as mean ± SD of three separate experiments. (*P < 0.05;**P < 0.01;***P < 0.001).

As shown in Figure 6, Rg1 reduces miR-23a expression in HUVECs. To confirm this effect of miR-23a on angiogenesis, we designed anti-miRTM miRNA Inhibitor (ASO-miR-23a) to knockdown expression of miR-23a. Both qRT-PCR and western blot analysis revealed that miR-23a reduces the expression of VEGF-A in HUVECs. Conversely, the addition of ASO-miR-23a increased VEGF-A expression (Figure 7).

### MiR-23a regulates cell viability and migration

**Figure 8:**
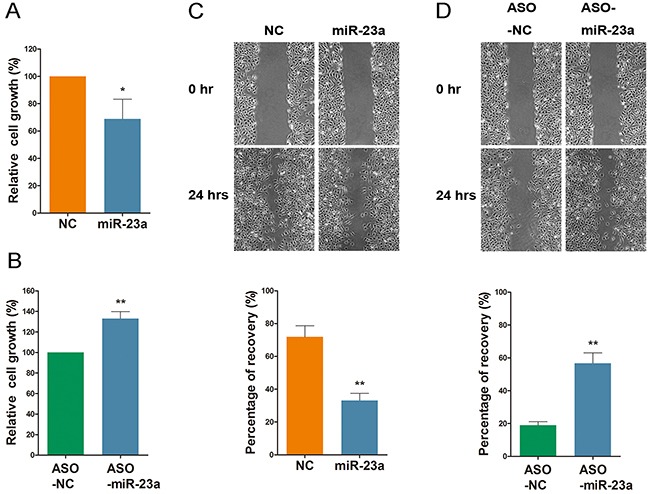
MiR-23a regulates cell viability and migration **(A** and **B)** Cell proliferation analysis following a 72 hour treatment with miR-23a or ASO-miR-23a. **(C** and **D)** HEK-293 transfected with miR-23a or ASO-miR-23a were seeded, and the artificial wounds were created. Pictures were captured at 0 and 24 h after scratching. The percentage of recovery were calculated. Data are plotted as mean ± SD of three separate experiments. (*P < 0.05;**P < 0.01).

To evaluate the function of miR-23a in cell growth and migration, HEK-293 cells were transfected with either miR-23a or treated with ASO-miR-23a. Overexpression of miR-23a resulted in a robust reduction of cell growth, and wound recovery decreased ~30%, as compared to the control group (Figure 8A and 8C). Moreover, the group treated with ASO-miR-23a expressed increased viability when compared with the control group. Furthermore, the wound recovery percentage increased from ~20% to 60% (Figure 8B and 8D). This suggests that miR-23a acts as a repressor of HEK-293 cell motility.

### MiR-23a regulates ginsenoside Rg1-induced angiogenesis *in vitro*

**Figure 9:**
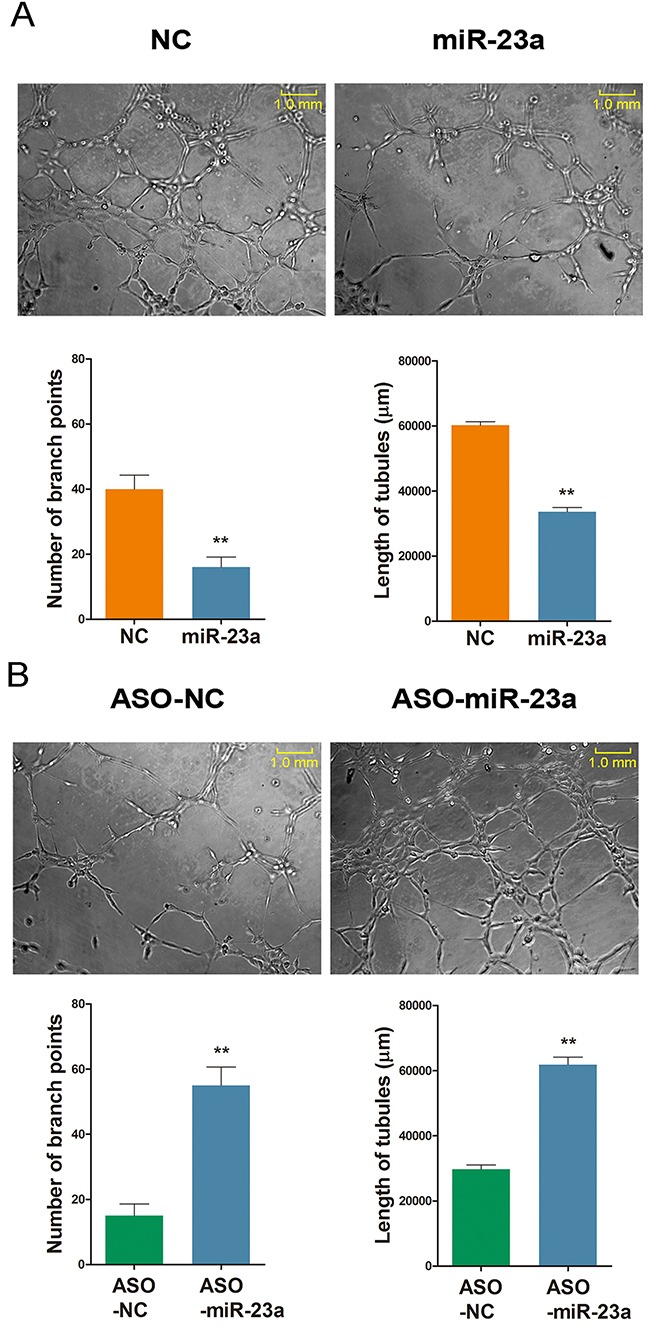
MiR-23a regulates ginsenoside Rg1-induced angiogenesis *in vitro* **(A** and **B)** Representative images demonstrating tube formation assay and statistical analysis. Cells stimulated with 250 nM Rg1 and transfected with either miR-23a or ASO-miR-23a on day 3. The number of branch points and the length of tubules were calculated. Data are plotted as mean ± SD of three separate experiments. (**P < 0.01).

The angiogenesic effect of miR-23a was examined using tube formation assay in HUVECs. There was a significant decrease in endothelial tube formation following miR-23a overexpression. The number of endothelial branch points decreased from ~40% to less than 20% when compared to the control group. However, transfection with ASO-miR-23a improved tube formation from less than 20% to about 55% (Figure 9).

### MiR-23a regulates angiogenesis in zebrafish

**Figure 10:**
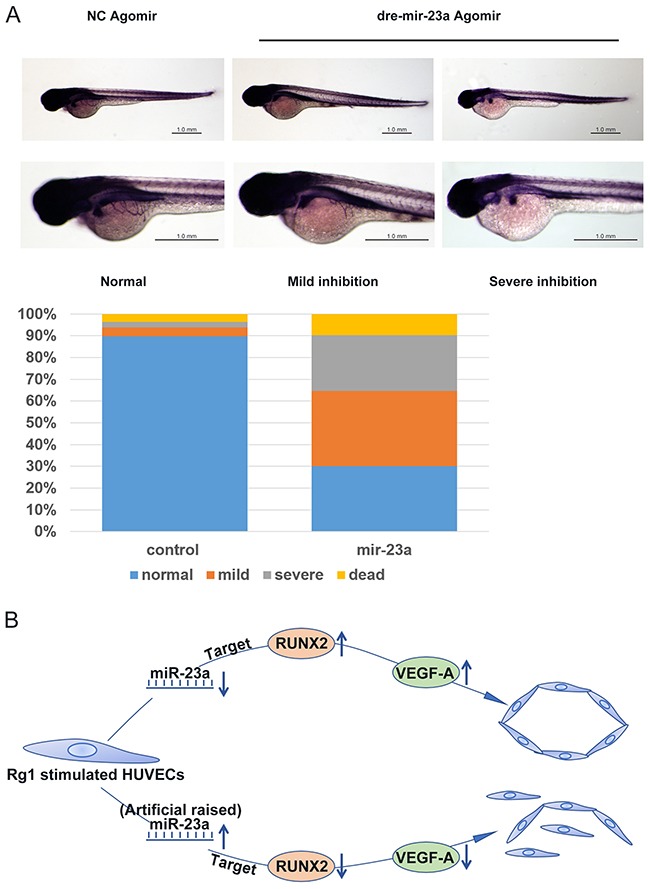
MiR-23a regulates ginsenoside Rg1-induced angiogenesis in zebrafish **(A)** Fertilized, one to four cell stage zebrafish embryos were injected with 10μM dre-mir-23a agomir or NC agomir. The SIV basket of miRNA-injected zebrafish embryos at 3 days post-fertilization (dpf) was stained by Alkaline Phosphatase Staining. For the miR-23a group, 297 zebrafish were observed, and for the control group, and 280 zebrafish were observed. **(B)** A graphical illustration of the molecular signaling events involved in MiR-23a/RUNX2/VEGF-A pathway for the regulation of HUVECs angiogenesis.

In order to identify an anti-angiogenic mechanism of miR-23a *in vivo*, zebrafish were utilized to perform an angiogenesis experiment [30]. Since agomir is more stable *in vivo* than miRNA, agomir was adapted for our experiments instead of the miRNA used in our previous *in vitro* studies. In this test, the dre-miR-23a agomir and its random sequence, NC agomir, were injected in zebrafish embryos. The formation of basket-like subintestinal vessels (SIV) was used to indicate the anti-angiogenic ability of miR-23a. We classified the ability to inhibit angiogenesis into three categories: normal, mild inhibition, and severe inhibition. We found that 90% of the control group zebrafish embryos were categorized as normal. However, in the dre-mir-23a group, only 30% of the embryos were normal, about 35% of embryos showed mild inhibition, and more than 20% of embryos were categorized as severe inhibition (Figure 10A). These data further confirm an anti-angiogenic function of miR-23a *in vivo*.

## DISCUSSION

Cancer is a major health concern because it is a leading cause of death worldwide. Statistics from 2016 estimate that over 1.6 million new cancer cases will occur in the United States [31]. Interestingly, ginseng showed a promising anti-cancer effect. Through the formation of conjugates with carbon nanotubes (CNT) and suppression of transforming growth factor-β1 (TGF-β1), Rg1 has shown a distinct anti-cancer effect in cancer cells [12, 13]. However, Rg1 also has an angiogenic function in endothelial cells, which may promote cancer development. Postnatal angiogenesis can only be initiated by angiogenic factors, such as VEGF-A and FGF, or receptors such as VEGF-A receptor. Endothelial cells remain quiescent and their turnover rates are in terms of months or years [32, 33]. Though the angiogenesis function of Rg1 in endothelial cells is advantageous for vascular disorders and injuries, it's disadvantageous for angiogenesis in the tumor microenvironment. Thus, we attempted to unearth a solution that can inhibit angiogenesis in endothelial cells located in the tumor microenvironment while maintaining the Rg1-induced anti-cancer effect in cancer cells.

It is widely accepted that miRNA has complicated roles in the regulation of different physiological and pathological processes. A single miRNA may differentially target multiple transcripts, while a single gene may be co-regulated by multiple miRNAs [34, 35]. Therefore, to fully comprehend a particular physiological and pathological process, emphasis should be put on the discovery of new miRNAs and their targets. Ample research has demonstrated that Rg1 may co-regulate different miRNAs and facilitate different key mechanisms of angiogenesis. These mechanisms include the expression of angiogenic factors NO and VEGF-A and Akt-mediated cellular survival pathways that induce angiogenesis. Moreover, artificial augmentation or suppression of endogenous miRNA levels can modulate pathological conditions. Such miRNA candidates provide biological techniques for normalizing gene expression in genetic disorders. The use of biological techniques may evade toxicity or drug resistance produced by turning a single target on or off [36]. Therefore, Rg1 may induce angiogenesis by modulating specific miRNAs. In our study, RUNX2 was identified as the target of miR-23a during Rg1-induced angiogenesis. The target region of miR-23a was confirmed as the binding site of RUNX2 transcript that extends from nucleotides 1061 to 1067 located within the RUNX2 3’-UTR. The inhibitory effect of miR-23a on RUNX2 3’-UTR was further confirmed in luciferase reporter gene assay. The interaction between miR-23a and its binding site in the 3’-UTR of the target RUNX2 gene transcript is important for activating target repression.

RUNX2 is an important biomarker associated with osteoblast differentiation whose mechanism has been demonstrated in several prior studies. For example, a previous study demonstrated that miR-2861 effects osteoblast differentiation through overexpression of RUNX2 protein in human periodontal ligament stem cells (hPDLSCs) [37]. Furthermore, RUNX2 also participates in cell cycle regulation. It has been reported that RUNX2 is a negative regulator of pRB (retinoblastoma protein), which has been demonstrated as a cell cycle inhibitor, thus promoting the cancer cell proliferation [38]. Recent studies have also shown that RUNX2 interacts directly with p53 and plays an opposing function when compared with p53[39, 40]. As a result, RUNX2 plays an important role in the process of cancer development. However, prior to our present investigation, few studies have addressed the mechanism of RUNX2 in angiogenesis. We discovered that RUNX2 expression is upregulated in Rg1-stimulated HUVECs. Furthermore, we demonstrated that VEGF-A expression is downregulated when si-RUNX2 was used and that RUNX2 is associated with cell viability, migration, and angiogenesis. This discovery addresses a novel function of RUNX2 and an innovative direction for treatment of anti-angiogenesis in cancer therapy. With RUNX2 identified as a potentially useful therapeutic target against cancer angiogenesis, we believe our findings will contribute to cancer therapy development.

In the current study, we established that a reduction in RUNX2 downregulates VEGF-A expression, while miR-23a reduced RUNX2 expression. However, the relationship between VEGF-A and miR-23a remains unclear. Therefore, gain-of-function and loss-of-function experiments were performed to clarify the relationship. When HUVECs were treated with miR-23a, VEGF-A expression was decreased. Since Rg1 reduced endogenous miR-23a levels in HUVECs, anti-miRTM miRNA Inhibitor (ASO-miR-23a) was designed to mimic the Rg1-induced decrease in miR-23a. When the ASO-miR-23a was administered, the expression of VEGF-A increased. These data are consistent with our proposal that RUNX2 expression is mediated by miR-23a. Therefore, we believe that Rg1-induced angiogenesis may be simulated through an increase in RUNX2 via the downregulation of miR-23a.

To investigate a potential mechanism, we examined the viability, migration, and tubulogenesis abilities following transfection with either si-RUNX2, miR-23a, or ASO-miR-23a. We found that viability, migration, and tubulogenesis were significantly increased when treated with ASO-miR-23a. Expectedly, we observed a decreased when transfected with si-RUNX2/miR-23a. Consistent with our hypothesis, overexpression of miR-23a decreased the translational activity of target gene (RUNX2) transcripts. These results are analogous to the knockdown of RUNX2, as they resulted in a decrease in tubular network formation in endothelial cells.

Additionally, our zebrafish model confirmed a regulatory role of miR-23a by detecting the formation of basket-like SIV in zebrafish embryos. These *in vivo* results further supported our conclusion and also confirmed that the function of miR-23a and RUNX2 won't lose efficacy under *in vivo* conditions. Thus, we identify RUNX2 as a promising potential target and present miR-23a as an effective strategy in anti-angiogenesis.

We presented a novel MiR-23a/RUNX2/VEGF-A pathway in ginsenoside Rg1-induced angiogenesis (Figure 10B). Moreover, the function of this novel molecular mechanism has been verified. Taken together, miR-23a targets RUNX2 and suppresses angiogenesis in Rg1-stimulated endothelial cells. This study provides a new solution to prevent cancer angiogenesis.

## MATERIALS AND METHODS

### Cell culture and chemical reagents

Human umbilical vein endothelial cells (HUVECs) and human embryonic kidney (HEK) 293 cells were purchased from American Type Culture Collection (ATCC). HUVECs were cultured in RPMI-1640 complete medium, and HEK-293 were cultured in DMEM (dulbecco's modified eagle medium) high glucose medium containing 10% FBS. All cells were cultured at 37°C in a humidified incubator with 5% CO_2_. Ginsenoside Rg1 was purchased from Sigma (68317, Sigma).

### Western blot analysis

Equal amounts of protein samples (20 mg) extracted from cells were separated by SDS-PAGE and transferred onto a nitrocellulose membrane. After blotting, the membrane was probed with primary antibodies against RUNX2 (Santa Cruz Biotechnology, USA), VEGF-A(abcam), and GAPDH(Sigma); and subsequently incubated with their appropriate secondary antibody. The membrane was then washed by TBST. GAPDH expression was used as protein loading control. Densitometry quantification was performed using Image J software. At least three independent experiments were carried out to study the protein expression.

### Quantitative RT-PCR based analysis of mRNA expression

Total RNA was extracted from HUVEC cells by using the E.Z.N.A.® Total RNA Kit II (OMEGA Bio-tek) and quantified using a NanoDrop 2000 (Thermo). A total of 100 ng of RNA from each sample was reverse transcribed using the PrimeScript™ RT reagent Kit (Takara). RUNX2 and VEGF-A mRNA was amplified by qRT-PCR using [RUNX2: forward, ACTTCCTGTGCTCGGTGCTreverse, GACGGTTATGGTCAAGGTGAA;VEGF-A:forward, TACCTCCACCATGCCAAGTG, reverse, GATGATTCTGCCCTCCTCCTT] and Takara SYBR Green II QPCR master mix in a Stratagene's MX3005P real time PCR machine. Data were normalized to GAPDH mRNA and then expressed as a percentage of control levels. The results were analyzed using the Wilcoxon signed-rank test.

### Cell migration assay

HEK-293 were plated onto a 6-well plates and incubated for 24 hours in RPMI1640 medium supplemented with 10% FBS. Mechanical scratching of the cell monolayer created an artificial wound. The wound in each well was captured using OLYMPUS microscope. Cells were then incubated for 24 hours and the bared area was captured again. Images at 0 and 24 hours were analyzed using Image J software. The migration of cells towards the wound was expressed as percentage of recovery.

### Cell transfection with anti-sense miRNA inhibitor

HUVECs were seeded onto 3.5 cm dish for 24 hours and subsequently transfected with anti-miRTM miRNA Inhibitor (ASO-miR-23a) (50 nM) (RIBOBIO, Guangzhou) using Lipofectamine^TM^ 2000 in Opti-MEM I Reduced Serum Medium (Invitrogen). Total RNA and cell lysate were collected for the indicated assays. Anti-miRTM miRNA Inhibitor Negative Control (ASO-NC) (RIBOBIO, Guangzhou) served as a negative control.

### Luciferase reporter gene assay

HUVECs were seeded (7 × 10^3^ cells/ well) in 96-well plates. According to the manufacturer recommended protocol, 100 ng of RUNX2 3’-UTR luciferase reporter plasmid (RIBOBIO, Guangzhou) and a mut RUNX2 miRNA mimic were transfected. All transfections were performed using Lipofectamine^TM^ 2000 in Opti-MEM I Reduced Serum Medium (100μl/well) for 24 hours. The luciferase activities from each well were measured using the Luciferase Assay System (Promega, USA) according to the manufacturer's instruction.

### Zebrafish embryos microinjection and vascular staining

Wild-type, natural pair-wised mature zebrafish were fed in fish tanks. Embryos were collected from the fish tank, and zebrafish at one to four cell stage were injected with 10μM dre-mir-23a agomir (AUCACAUUGCCAGGGAUUUCCA) or NC agomir (UUCUCCGAACGUGUCACGUTT) (Sangon Biotech, Shanghai). Each embryo was injected 0.03 pmol in total. Three days after fertilization, zebrafish embryos were fixed in 10% formalin. Then all zebrafish embryo SIV basket blood vessels were stained with nitroblue tetrazolium/5-bromo-4-chloro-3-indolyl-phosphate (NBT/BCIP) (Roche) at room temperature under dark conditions. Finally, the stained zebrafish were observed and photographed under stereomicroscope (OLYMPUS SZX16).
